# Uncalibrated Single-Camera View Video Tracking of Head Impact Speeds Using Model-Based Image Matching

**DOI:** 10.1007/s10439-025-03705-2

**Published:** 2025-03-13

**Authors:** Nicole E.-P. Stark, Ethan S. Henley, Brianna A. Reilly, John S. Nowinski, Gabrielle M. Ferro, Michael L. Madigan, Damon R. Kuehl, Steve Rowson

**Affiliations:** 1 Department of Biomedical Engineering, Virginia Tech, 440 Kelly Hall, 325 Stanger Street MC 0298, Blacksburg, VA 24061 USA; 2 Department of Electrical and Computer Engineering, Virginia Tech, Blacksburg, USA; 3 School of Neuroscience, College of Science, Virginia Tech, Blacksburg, VA USA; 4 ICTAS Institute for Critical Technology and Applied Science, Blacksburg, VA USA; 5 Department of Industrial and Systems Engineering, Virginia Tech, Blacksburg, VA USA; 6 Department of Emergency Medicine, Virginia Tech Carilion, Roanoke, VA USA; 7 Emergency Medicine, Virginia Tech Carilion School of Medicine, Blacksburg, VA USA

**Keywords:** Head impacts, Model-based image matching, Motion tracking, Videogrammetry

## Abstract

**Purpose:**

This study evaluates the accuracy of a model-based image matching (MBIM) approach with model calibration for tracking head impact speeds in uncalibrated spaces from single-camera views.

**Methods:**

Two validation datasets were used. The first included 36 videos of guided NOCSAE headform drops at varying camera positions (heights, distances, camera angles) where a speed gate measured vertical impact speed. The second dataset had eight videos of participants performing ladder falls with marked helmets, captured using a 12-camera motion capture system to track head impact speeds. Each video was tracked frame-by-frame, matching a 3D NOCSAE headform model to the head using MBIM software. Accuracy was assessed by comparing captured to MBIM-tracked speeds by the mean difference and Root Mean Square Error (RMSE). A linear model assessed the influence of camera position.

**Results:**

For ideal camera views (90 degrees, height 1 or 1.4 m), MBIM-tracked vertical speeds were 0.04 ± 0.15 m/s faster than the true speed (RMSE 0.15 m/s; 2.3 ± 6.2% error). Across all 36 NOCSAE videos, MBIM-tracked vertical speeds were 0.03 ± 0.19 m/s faster (RMSE 0.19 m/s; 1.8 ± 6.9 % error). In participant videos, MBIM-tracked resultant speeds were 0.01 ± 0.33 m/s slower (RMES 0.31; 0.7 ± 9.5% error) compared to motion capture.

**Conclusion:**

MBIM with model calibration can analyze head impact kinematics from single-camera footage without environment calibration, achieving reasonable accuracy compared to other systems. Analyzing head impact kinematics from uncalibrated single-camera footage presents significant opportunities for assessing previously untraceable videos.

**Supplementary Information:**

The online version contains supplementary material available at 10.1007/s10439-025-03705-2.

## Introduction

To best understand head injury biomechanics in sports, car accidents, falls, and other environments, the tracking and analysis of the head impact kinematics is crucial [1]. Capturing head impact kinematics also guides the development of protective equipment, testing protocols, and injury prevention strategies [1–6]. Videogrammetry offers a great option for tracking head impact kinematics captured on video [7]. However, these analyses have been conducted in calibrated environments and supported by multiple-camera views to accurately capture head impacts from videos [8–15]. Yet, numerous head impacts are recorded using only a single camera, often without any accompanying calibration information. This presents a significant challenge to extract impact kinematics from such videos due to the absence of calibration data or additional camera perspectives, limiting the depth of analysis possible from these common yet critical recordings.

Videogrammetry tracking techniques such as point-click, Kinovea, and model-based image matching (MBIM) have been used to extract head impact kinematics from videos in various settings. Hendricks et al. and Choi et al. both used a point-click tracking method and environment calibration, field lines, or a 25-point grid, to convert pixels to meters [9, 16]. Hendricks et al. had reasonable success with a 0.11 ± 0.30 to 0.62 ± 0.57 m/s mean difference (± 95% limits of agreement), from a 1.29 to 14.6 m/s captured velocity range between their tracking methods and the standard calculated velocity [16]. Choi et al. used grid calibration and obtained a mean difference (± SD) of 0.06 ± 0.21 to − 1.01 ± 0.55 m/s between the tracked and motion capture vertical velocity that ranged from 1.29 to 3.51 m/s, dependent on the fall direction [9]. Kinovea software has also been validated for tracking head impact kinematics, showing a 4% to 11% error in head impact velocity tracking [10, 11]. However, Kinovea still requires calibration with field lines, grid, or subject height, which may not always be readily available for videos captured outside controlled environments or without the possibility of post-recording site visits.

MBIM techniques have been used to track head impact kinematics and gait mechanics with reasonable success [12, 17]. Tierney et al. used Poser 4 and Poser Pro Pack with a model skeleton and surrounding environmental measures to calibrate the cameras [12]. They achieved the best results with multi-camera views, producing an error of 0.42 ± 0.07 m/s to 1.29 ± 0.21 m/s (RMSE ± SD) from a captured range of velocities from − 10.82 to 1.23 m/s. However, Poser 4 is not available on the market, and the current version of Poser is optimized for 3D animation rendering. MBIM has also been implemented in other environments outside of Poser 4. Bailey et al. and Jadischke et al. both used 3D scans to create a calibrated space and had a 9% (0.4 m/s) and 10.7% (0.24 m/s) error in their MBIM technique [14, 15]. However, both methods required 3D scans of the environments to calibrate the space.

Overall, validation studies of videogrammetry reveal notable variance in accuracy across methods in the literature. These systems have been implemented to extract head impact speeds from 2D footage, but they all require a calibrated environment and multiple-camera views. This limits the analysis of real-world incidents captured by single-camera views in uncalibrated spaces. Obtaining high-quality multi-view perspective and calibration information is rare unless the impact occurs within a professional or research setting already equipped for calibration. Additionally, conducting post-capture calibration demands access to the original environment of the video capture, a requirement often unmet for footage recorded ‘in the wild’ or outside controlled settings. Consequently, numerous videos captured in such uncontrolled environments elude detailed analysis for head impact kinematics.

MBIM with model calibration offers a promising solution to tracking single-camera footage without environmental calibration. MBIM involves projecting a scaled 3D model onto a 2D video frame. MBIM with model calibration makes it possible to track head impact kinematics from 2D single-camera recorded videos in uncalibrated space with reasonable accuracy, opening opportunities to capture a wide range of head impact kinematics from different settings.

This study aims to evaluate the accuracy and reliability of an MBIM method using model calibration to track head impact speeds using a single-camera view against controlled headform drop tests and participant head impacts during falls. We set three main objectives: first, to assess the MBIM method’s accuracy under optimal camera views, low viewing heights, and camera tilt angles perpendicular to the fall’s plane. Our second objective was to determine the accuracy of the MBIM method across camera-varying views, including varying distances, camera tilt angles, and the camera’s height from the impact. Our third objective was determining the interrater reliability of capturing head impact kinematics using the MBIM method. These results provide a basis for understanding how accurately head impact kinematics can be captured using an MBIM approach with only model calibration and a single-camera view. Furthermore, the successful implementation of MBIM with model calibration has the potential to improve our understanding of head kinematics by capturing impact videos from a wide range of environments.

## Materials and Methods

We used two validation datasets to evaluate the accuracy of the MBIM with model calibration using a single camera. The first set comprised 36 video recordings of guided drop tests using a NOCSAE headform at two vertical speeds, confirmed with a speed gate. These video recordings also varied between three camera heights, three camera distances, and from different camera angles. The second set consisted of eight video recordings of ladder falls in which participants performed backward and side falls, captured with a motion capture system (Qualisys North America, Inc.) and video recordings. Each video from both datasets was tracked frame-by-frame in a MBIM software developed in godot (version 4.2.2). This software virtually posed a to-scale 3D model of a NOCSAE headform onto the video frame. The headform’s position, rotation, and scale were manually manipulated to match the projected headform to the 2D video frame, and the impact speed was calculated based on two frames and the frame before impact. This approach ensures that movement is measured consistently over the entire time interval used to calculate velocity. We evaluated the accuracy of the MBIM system by comparing observed (speed gate or motion capture) speeds and the MBIM-tracked speeds by calculating the mean difference and standard deviation (SD) and calculating Root Mean Square Error (RMSE). We also used a linear model to evaluate the influence of camera position on the accuracy of tracked vertical impact speed.

### NOCSAE Guided Drop Tower Dataset

**Fig. 1:**
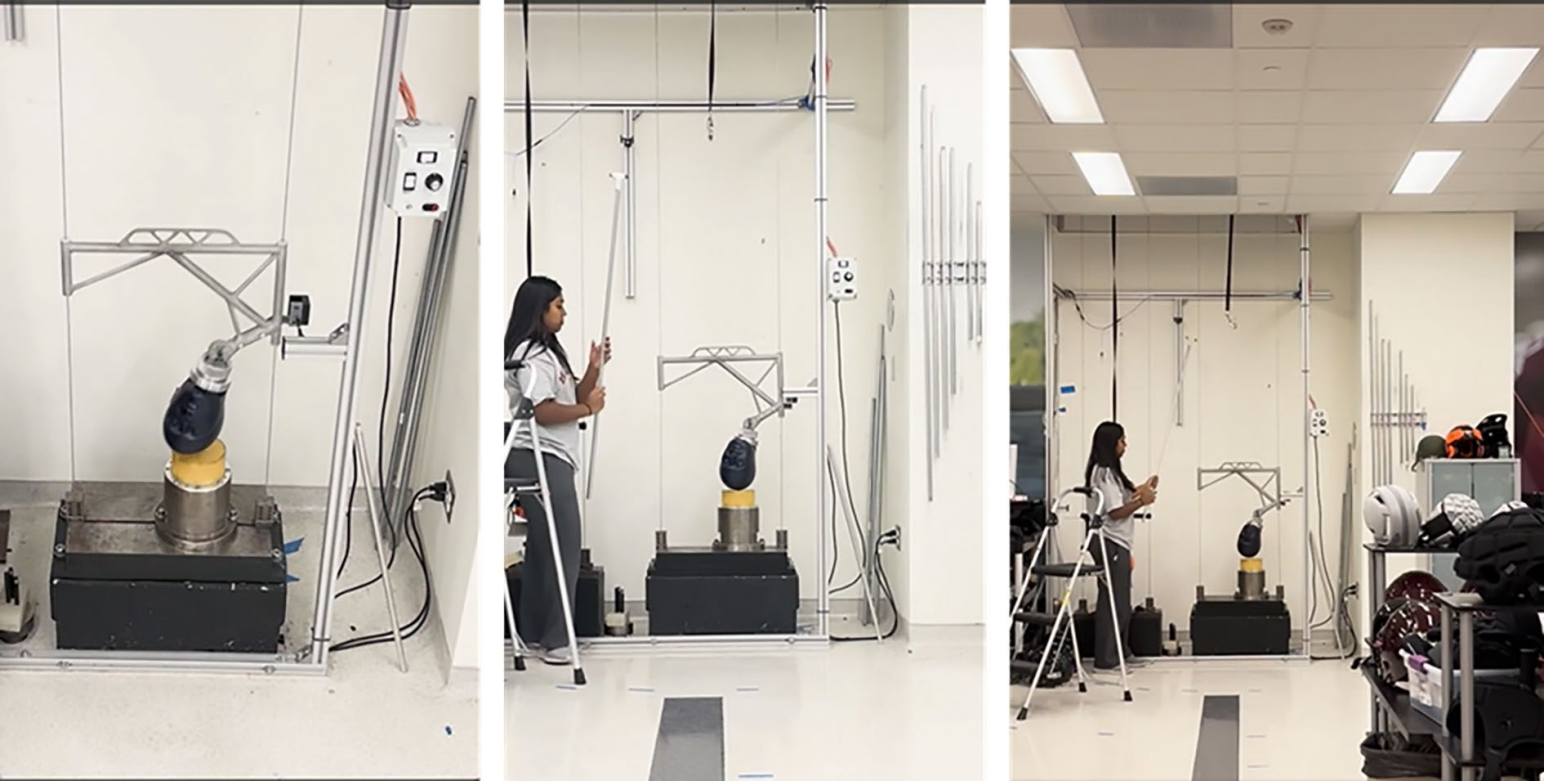
Guided NOCSAE drop tower taken from different camera positions where observed speeds were captured with a speed gate and recorded. The left image was taken from a 2.8 m height, 3 m distance, and 55-degree camera angle. The middle image was taken from a 1.4 m height, 6.1 m distance, and 75-degree angle. The right image was taken from a 1 m height, 9.1 m distance, and 90-degree angle. Each drop was video-recorded for MBIM tracking

36 guided drop tests using a NOCSAE headform (Fig 1) were captured on video recording (30 fps, 1080 p), and observed speeds were confirmed with a speed gate for each impact. These impacts were recorded from four heights from the ground (1, 1.4, 2.5, 2.8 m), where the impact occurred at 0.7 m above the ground and three distances from the impact center (3, 6.1, 9.1 m) (Table 1). These heights were chosen to represent a small ground-mounted tripod (1 m), a person capturing a video with their phone (1.4 m), and a ceiling height typical of surveillance footage (2.8 m from 3 & 6.1 m distance; 2.5 m for 9.1 m distance). At the ground-mounted height (1 m), the camera angle was only tested at 90 degrees relative to the ground perpendicular to the impact. Tests at a camera height of 1.4 meters were conducted with the camera tilted at angles of 90 degrees and 75 degrees. The camera angles were measured relative to the ground, where 90 degrees was perpendicular to the surface, and 75 degrees represented a 15-degree tilt toward the point of impact. For the ceiling-mounted height (2.8 m), at the 3 m distance, we tested at three camera angles (45, 55, 65 degrees), and at the 6.2 m distance, we tested at three camera angles (55, 56, 75 degrees). While at the 9.1 m distance and the ceiling height, we tested two camera angles of 65 and 75 degrees (Table 1). These were determined based on the field of view that captured the drop from the given distance and height. Each impact was conducted at two drop speeds, 2 m/s and 4 m/s, for a total of 36 captured impacts.

**Table 1:** Camera position descriptions by height, including distance and angles, for capturing the NOCSEA guided drop tower dataset

Height (m)	Distance (m)	Angles (degrees)
1	3	90
	6.1	90
	9.1	90
1.4	3	90, 75
	6.1	90, 75
	9.1	90, 75
2.5	9.1	65, 75
2.8	3	55, 55, 65
	6.1	45, 55, 65

### Participant Falling Dataset

The second dataset consisted of eight video recordings (30 fps) of ladder falls approved by Virginia Tech Institutional Review Board (22-089, date 3/4/22) [18]. The observed head impact kinematics were captured with a 12-camera motion capture system (Qualisys North America, Inc., Buffalo Grove, IL). Participants included three young adults (1 female) with a mean body height of 175 ± 13 cm and body mass of 80 ± 16 kg. Exclusion criteria included any self-reported musculoskeletal injuries within the past six months, concussion in the past five years, pregnancy, excessive fear of falling, concerns with bruising, body mass greater than 100 kg, or height greater than 1.8 m. Participants wore a hockey helmet (Covert PX + , Warrior Sports Inc., Warren, MI) with the chin strap fitted according to the manufacturer’s recommendations and wrist braces. Four markers were applied to each participant’s helmet, all in a horizontal plane 3 cm superior to the front edge of the helmet, with markers on the anterior and posterior aspects and above each ear. To mitigate injury, the fall area (1.3 m x 2.9 m) was covered in 15-cm-thick padding comprised a cheer mat (Cheer Sting Mat, Mancino Manufacturing Co. Inc., Lansdale, PA) on top of a rock-climbing crash pad (Magnum Crash Pad, Metolius Climbing, Bend, OR).

**Fig. 2:**
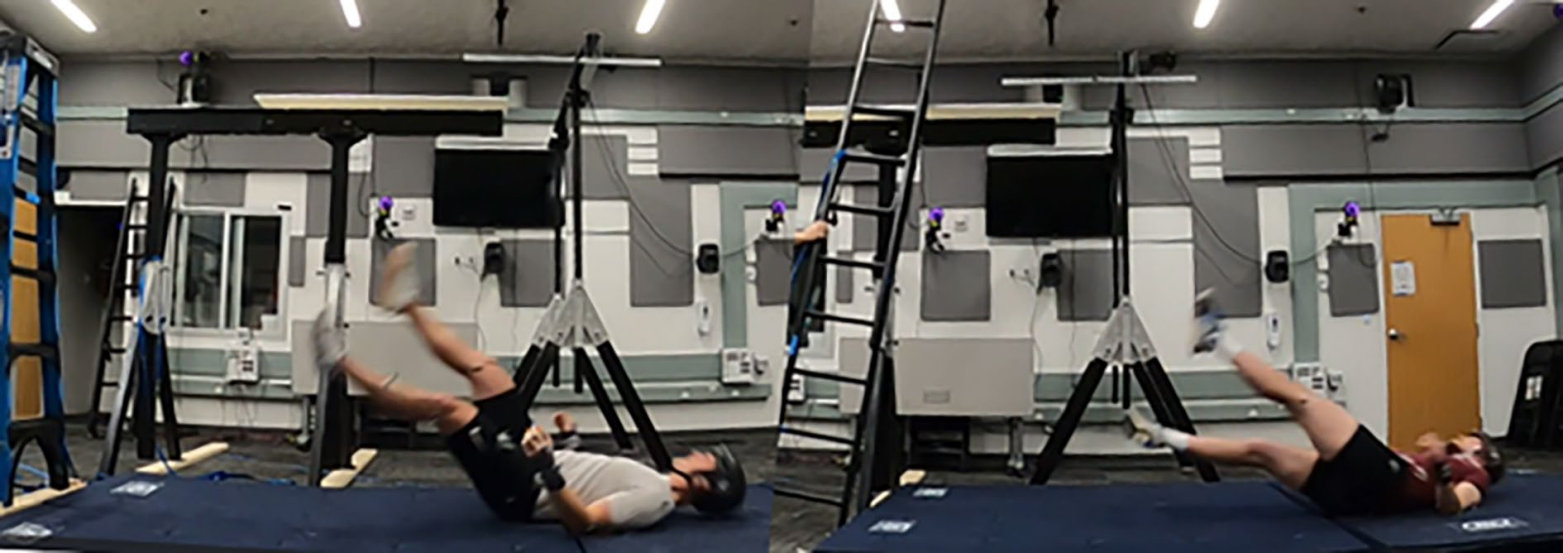
Participant ladder falls showing head impacts. The participant falls were not restricted. Each fall was captured with video and a 12-camera motion capture system

Each participant completed four falls and was instructed not to resist a loss of balance or a fall to the ground if it occurred (Fig 2). Two falls were completed on a step ladder where participants reached overhead and manipulated a puzzle cube from two heights, 1.4 m and 1.8 m, to mimic overhead work. Simultaneously, an investigator provided a firm backward push to the ladder to simulate the ground sinking under the ladder feet to generate the fall. Two falls were also completed on an extension ladder where participants leaned sideways to push a ball on an aluminum bar parallel to the beam and slightly above the participant’s shoulder height as far as possible away from themselves while maintaining their balance on the ladder, at two heights 1.1 m and 1.7 m. An investigator provided a firm sideways push to the ladder while the participant reached laterally to simulate the ground sinking under the ladder feet to generate the fall. Participant falls were not restricted, head impacts happened organically, and each was recorded from the same position.

Only falls with a clear head impact on video were used in this study, resulting in eight evaluated falls. This included four falls off the step ladder, two at 1.4 m and two at 1.8 m, and four falls off the extension ladder, two at 1.1 m and two at 1.7 m. Head impact kinematics were sampled at 128 Hz through the 12-camera motion capture system. Marker data was filtered at 14 Hz (fourth-order zero-phase-lag Butterworth), and we calculated vertical and horizontal impact speeds based on the head center of mass approximated as the average of the four helmet-mounted markers, taken 7 ms before impact (one sample before impact).

### MBIM Tracking

We developed an MBIM program in-house using the godot game engine (version 4.2.2). A 3D to-scale model of the NOCSAE headform was imported into Blender (version 4.0), and headform measurements were verified between the model and the physical dimensions. This model was imported into the godot framework with preserved dimensions such that the headform was calibrated to the 3D space in the game engine (2). The MBIM program was designed to manipulate the headforms’ position, scale, and rotation in the 3D space to match that seen on a projected 2D video frame-by-frame. A Blender model of the NOCSAE headform with a matched helmet was used for the participant ladder fall videos.

**Fig. 3:**
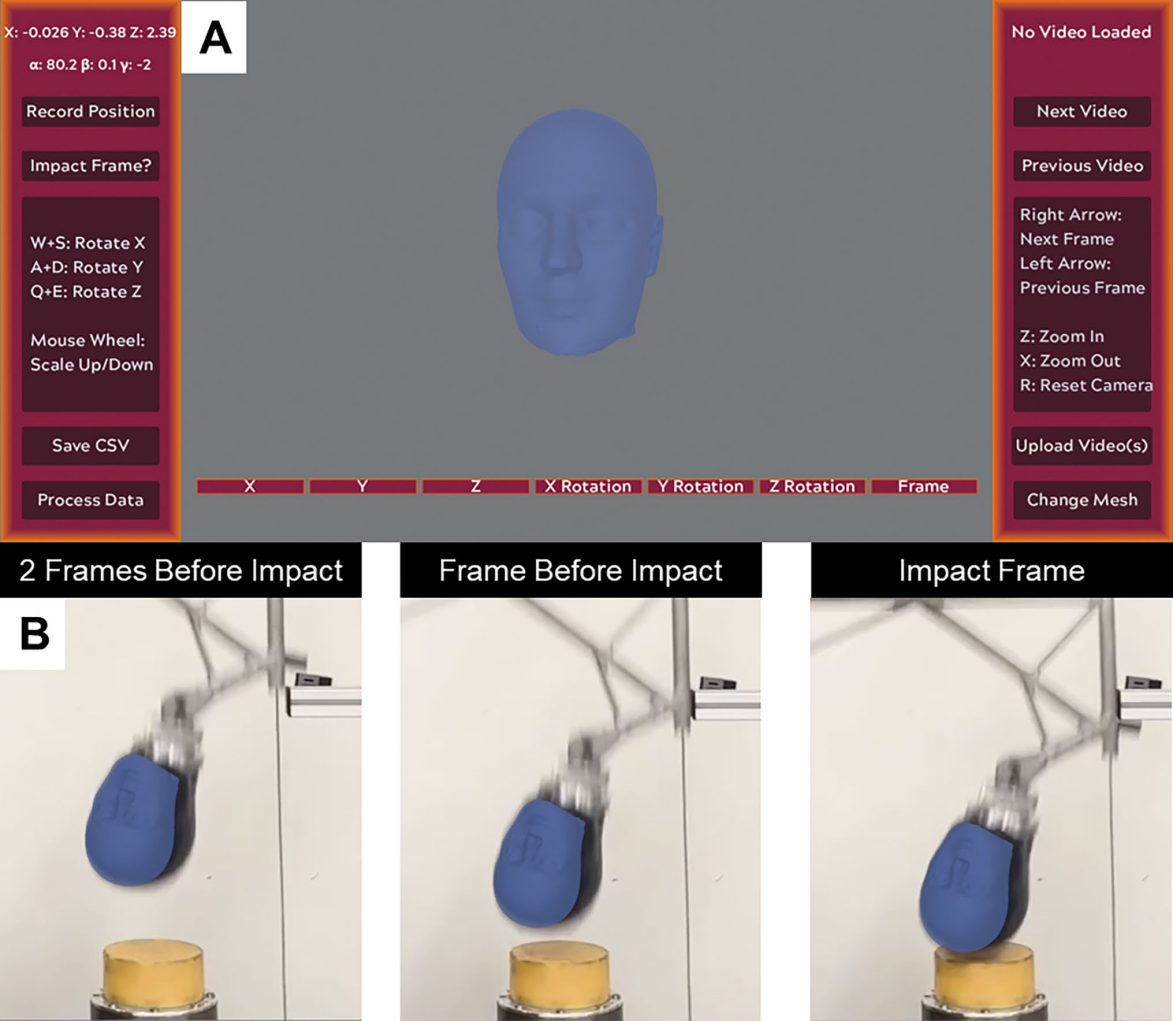
The MBIM program user interface developed on the godot Game Engine used to track 2D videos frame-by-frame. **a** The imported-to-scale NOCSAE headform in unity framework. **b** Tracked MBIM linear drop tower NOCSAE video: two frames before impact, one frame before impact, and impact frame. Vertical impact speed was calculated from two frames before impact and one before impact*.* Detailed MBIM processing procedure is included in the supplemental material

Each video from the collected datasets was imported into the MBIM program (Fig 3). Matching the head of a known size to each video enabled the calibration of the frame to real-world coordinates (pixels to meters). The headform was then manually matched and tracked frame-by-frame to the 2D video across six degrees of freedom, including matching position, rotation, and scale by two individual trackers. The impact frame was also identified and captured in each track. Each tracker underwent a structured training process that included detailed instructions and practice sessions using representative videos. Trackers were instructed to use the zoom feature to focus on the head/headform being tracked, enlarging it in the software. Then, use the macro adjustment features to best match the head/headform position and scale. This was followed by the micro-adjustment feature to precisely control the matching across six degrees of freedom. The alignment quality was self-assessed based on minimizing placement and sizing discrepancies of headform features (e.g., edges of headform, nose, eyes, mouth). Detailed MBIM processing procedure is included in the supplemental material.

We then calculated the vertical (perpendicular to the impact surface), horizontal (parallel to the impact surface), and depth (depth from camera perspective) head impact speeds as the change in position over time between two tracked frames. Impact velocities were calculated between the frame two frames before impact and the frame before impact. The MBIM software recorded the scale of the sized headform and the pixel positions of the tracked frames. Using the known size of the head and scale factor enabled the calibration of pixels to meters. The MBIM software also reads the video’s frame rate. Velocity was then calculated by converting pixels to meters through the scaled headform, and the frame rate of the video. The resultant velocity from the vertical, horizontal, and depth was also calculated for the participant falls. Each video was repetitively tracked until the standard error of the tracked speed was under 0.25 m/s. To ensure mitigate operator bias, trackers were blinded to their tracking results, and an external reviewer would indicate if additional tracks were needed to reduce the standard error between tracks to under 0.25 m/s. Videos were tracked on average 2.9 ± 1.0.

### Statistical Analysis

MBIM system accuracy was measured by comparing the MBIM-tracked speeds to the recorded NOCSAE guided drop tower speed gait or motion capture speeds. We calculated the mean, standard deviation, and RMSE between the observed and MBIM-tracked speeds. All statistical analyses were completed in R (Version 4.3.3, RStudio; Boston, Massachusetts, USA). Camera position effect on tracked vertical impact speed for the NOCSAE guided drop tower videos was evaluated using a linear model, with a significance level of α < 0.05. The linear model compared the effects of camera height, camera distance, and camera angle on the difference between the speed gate and the MBIM-tracked vertical impact speed. A linear model was also fitted between MBIM-tracked impact speed and the speed gate to evaluate the influence of both camera angle and height.

Two raters tracked the 36 NOCSAE guided drop tower videos. This comparison was made due to the subjective nature of aligning and sizing the headform in the MBIM program with the head in the video. Intraclass Correlation Coefficients (ICC) were used to compare the tracked vertical impact speed between the two raters.

## Results

### NOCSAE Drop Tower & Effect of Camera Angle

**Fig. 4:**
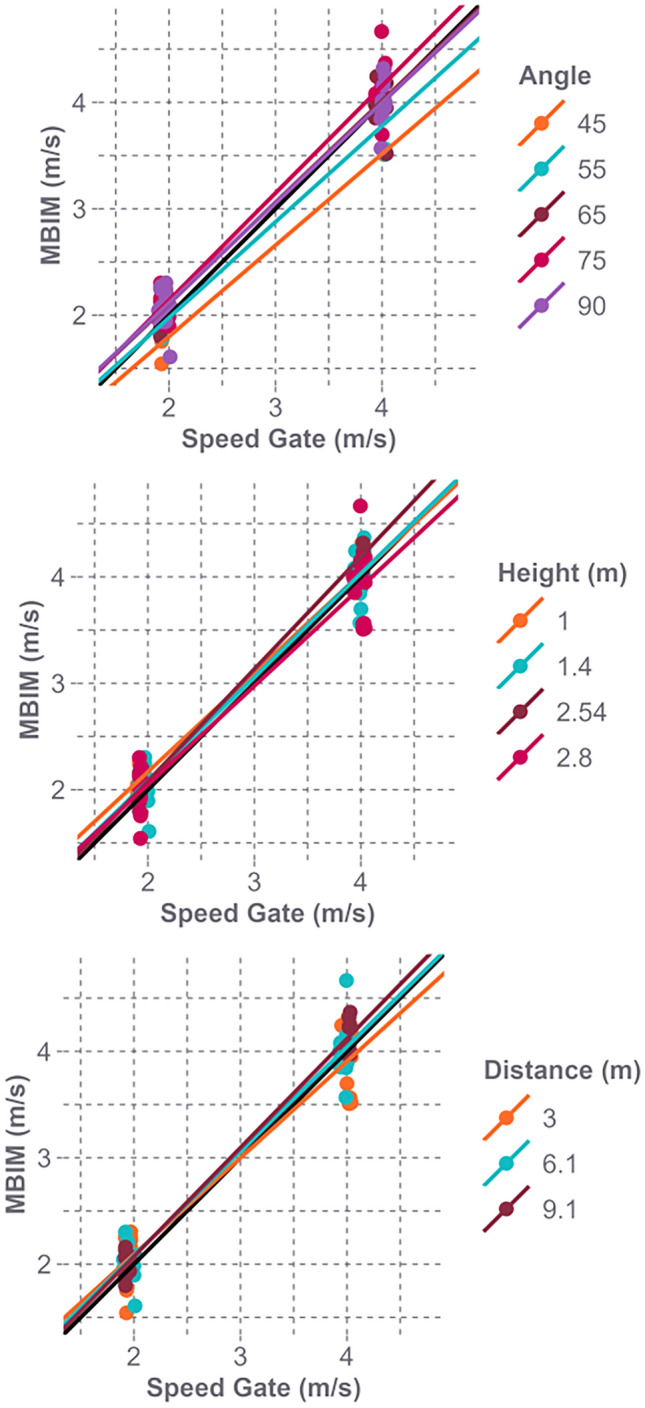
Difference between the observed and MBIM-tracked impact speed evaluated by camera position. As the camera tilt angle decreased, there were a larger negative difference between the observed and MBIM-tracked vertical impact speed

The average impact speed recorded by the speed gait for the NOCSAE headform drops was 2.0 m/s and 4.0 m/s. Comparing ideal camera views (90 degrees camera tilt angle and height of 1 or 1.4 m), the MBIM-tracked vertical speeds were 2.3 ± 6.2% (0.04 ± 0.15 m/s) faster (mean difference ± SD) than the observed, speed gate speeds (RMSE 0.15 m/s). Across all 36 NOCSAE headform drop videos and ideal and non-ideal camera views, the tracked speed had similar errors (Fig 4). Across all videos, the MBIM vertical speeds were 1.8 ± 7.0% (0.03 ± 0.19 m/s) faster than the observed speed (RMSE 0.19 m/s).

The analysis revealed that the accuracy of tracking vertical speed decreased as the camera angle increased (p = 0.009), indicating less accuracy as the camera tilt angle decreased (Table 2). Conversely, the distance of the video capture (p = 0.896) and the height of the camera (p = 0.809) did not affect the accuracy of the tracked vertical speed (Fig 4).

**Table 2:** The slope (m) and intercept (b) for the regression fit lines between the MBIM track velocity and the speed gate velocity (y = m*x + b) based on camera angle and height

Angel (degree)	Height (m)			
	1	1.4	2.5	2.8
45	NA	NA	NA	0.86; − 0.08
55	NA	NA	NA	0.91; − 0.16
65	NA	1.00; − 0.12	1.09; 0.27	0.86; − 0.47
75	NA	1.00; − 0.05	1.01; − 0.21	0.97; − 0.34
90	0.93; − 0.31	0.94; − 0.20	NA	NA

A perfect linear fit is represented by m = 1.00 and b = 0.00

### Inter-Rater Reliability

The mean difference between the two raters’ MBIM-tracked vertical speed was 0.27 ± 0.21 m/s, and the mean RMSE between the two raters was 0.35 m/s. Furthermore, the ICC was 0.94 (95% CI: 0.88 – 0.97).

### Participant Ladder Falls

**Fig. 5:**
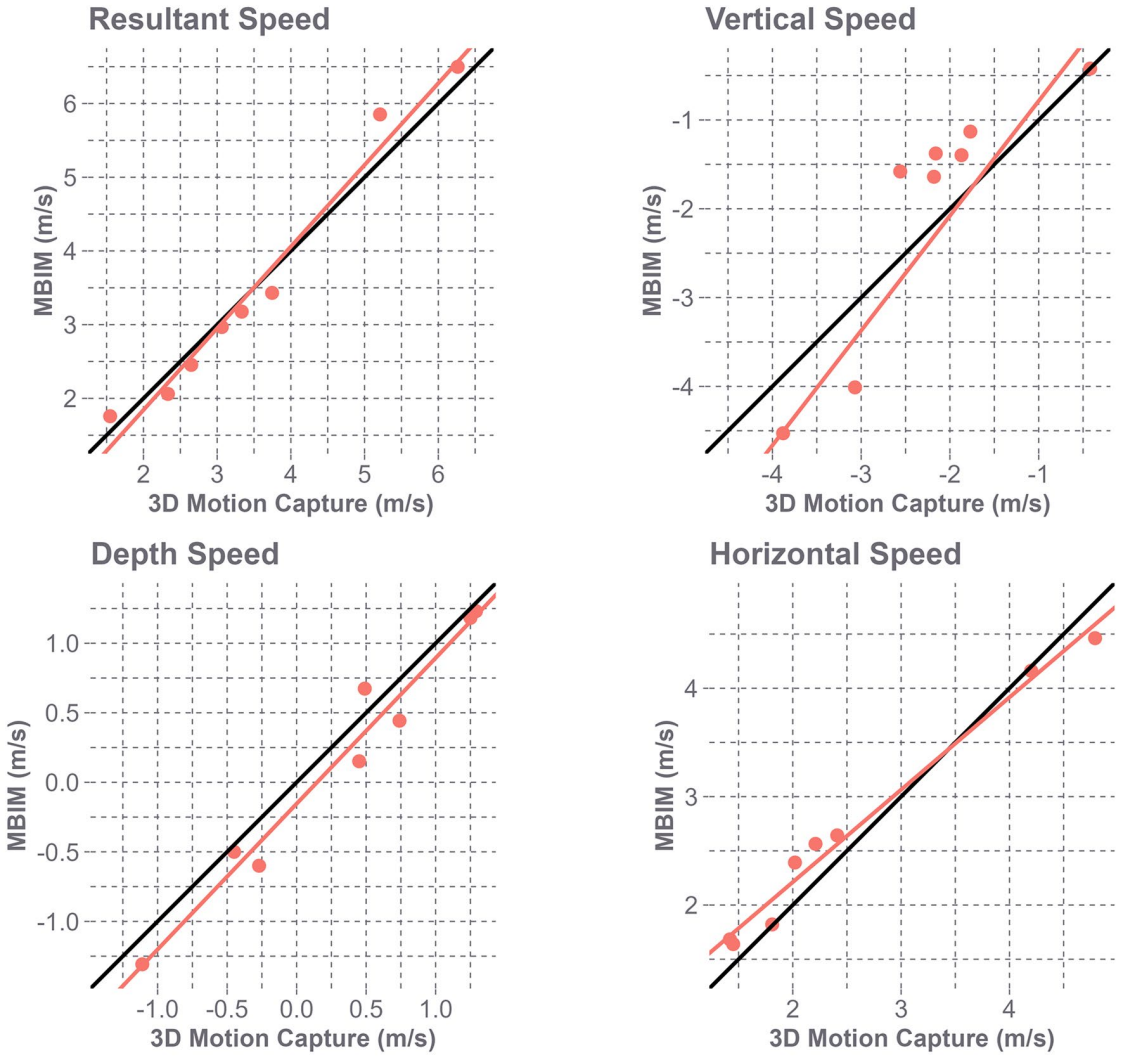
A comparison of the participant ladder falls between the MBIM-tracked and the observed 3D motion capture, resultant, vertical, horizontal, and depth speeds. The orange line is the fit regression between the MBIM-tracked and 3D motion capture speeds, and the black line is the ideal fit model

MBIM-tracked resultant speeds were 0.7 ± 9.5% slower than the 3D motion capture resultant speed (− 0.01 ± 0.33 m/s; RMES 0.31) (Fig. 5). Overall, the ladder fall head impacts captured by 3D motion capture had a range of vertical speed − 3.88 – − 0.42 m/s, horizontal speed 1.42 – 4.79 m/s, and depth speed − 1.11 – 1.29 m/s. On average, MBIM-tracked vertical speed was 0.22 ± 0.69 m/s slower (RMSE 0.69 m/s), horizontal speed was 0.13 ± 0.24 m/s slower (RMSE 0.26 m/s), depth speed was 0.14 ± 0.17 m/s faster (RMSE 0.21 m/s) compared to observed speeds (Fig 5).

## Discussion

In this study, we determined the accuracy of using MBIM with model calibration and a single-camera view for estimating head impact speeds compared to laboratory-based headform drops measured using a speed gate and against falls captured with 3D motion capture. Additionally, we investigated the MBIM with the model calibration method’s accuracy across varying camera positions, considering differences in distances, camera tilt angles, and camera heights. The MBIM with model calibration demonstrated the capability to track head impacts accurately and had high interrater reliability. The MBIM method’s ability to track head impacts in 2D single-camera view videos within an uncalibrated environment, with higher accuracy to more complex methods requiring calibration or multiple-camera angles. This MBIM method offers a vast opportunity to capture head impact kinematics.

MBIM with model calibration method’s estimation of vertical impact velocities from laboratory-based drops exhibited a 1.8 ± 7.0% error, translating to a speed deviation of 0.03 ± 0.19 m/s and an RMES of 0.19 m/s. This discrepancy between the observed speeds, speed gate, and the speeds tracked with MBIM is attributed to challenges in manually scaling the headform to match the video frame. Sizing adjusts the pixel-to-meter ratio, artificially lowering or increasing the tracked speed compared to the actual impact velocity. The primary sources of baseline errors are likely twofold: human operators’ inaccuracies in aligning the headform with the video frame and limitations imposed by the video resolution.

Ideal camera angles for capturing head impacts in video recordings are rare, highlighting the importance of considering how capture angle, height, and distance affect tracking accuracy. The accuracy of MBIM in tracking vertical impact speeds was influenced by the camera tilt angle. Our findings revealed that steeper camera angles led to greater errors in tracked MBIM speeds: at a 45-degree angle, the error was 10.7 ± 7.1%, while at a 90-degree angle, it reduced to 3.0 ± 8.0%. This trend suggests that steeper camera angles compromise tracking accuracy. However, camera height and distance had minimal effect on tracking accuracy. Steeper camera angles, such as those commonly found in ceiling-mounted security camera systems, often introduce greater out-of-plane motion, impacting measurement accuracy. Therefore, when using the MBIM method, careful consideration should be given to both the camera angle and the height of capture to optimize tracking accuracy. Distance between the camera and the impact event had a minimal effect on error, possibly because greater distances tend to align the camera angle more orthogonally to the impact event. This evaluation was limited, as our analysis focused solely on the impact of camera positioning on vertical, not horizontal, impact speed tracking. Prior research has shown that a 90-degree angle optimizes the tracking of horizontal kinematics, but the camera angle, rotation around the impact, does not affect vertical impact speed tracking [9]. When tracking horizontal impact speeds that are out-of-plane of the camera, similar horizontal tracking errors are expected to those of the vertical ones. However, this future work should evaluate horizontal tracking errors with camera position.

Human falls are more complex than controlled laboratory-based drops, encompassing both vertical and horizontal movements and often involving unpredictable translations in and out of the camera’s depth of field. Consequently, we assessed the accuracy of this MBIM method in capturing participant falls from a ladder. MBIM with model calibration demonstrated similar accuracy in tracking human falls, recording an average error of 0.7 ± 9.5% for resultant velocity. Although MBIM with model calibration has less accuracy at tracking velocity components, recording an average error of 0.22 ± 0.69 m/s for vertical speed, 0.13 ± 0.24 m/s for horizontal speed, and 0.14 ± 0.17 m/s depth speed. The diminished accuracy in vertical velocity is primarily attributed to the challenges in tracking movements within the depth plane of the camera.

Comparing the MBIM model calibration with a point-click method that uses field lines for calibration, the MBIM tracking of head impact speed performed better. The point-click methods reported a mean difference ± limits of agreement of 0.11 ± 0.30 to 0.62 ± 0.57 m/s for a tracked range of velocity from 1.29 – 14.6 m/s, while MBIM with model calibration mean difference and SD was 0.01 ± 0.33 m/s over 1.55 – 6.26 m/s velocity range (0.7 ± 9.5% error) [16]. Additionally, MBIM with model calibration performed better than another point-click method that used a 25-marker panel for calibration and a single-camera view (mean difference ± SD of − 0.06 ± 0.21 m/s to − 1.01 ± 0.59 m/s over a 1.29- 3.51 m/s range [9]) but had similar accuracy as the Kinovea method (0.03 ± 0.15 to − 0.19 ± 0.18 m/s with a less than 9% error [10]) using the same calibration approach. However, against the Kinovea method calibrated with participant height, the MBIM method with model calibration was more accurate in tracking horizontal velocities and vertical velocities [10]. MBIM with model calibration outperformed MBIM using Poser technology, which demonstrated an error range of 0.42 ± 0.07 to 1.29 ± 0.21 m/s over a − 10.89 to 1.23 m/s range [12]. Additionally, when compared to the work of Bailey et al. and Jadischke et al., who utilized MBIM with 3D scans for spatial calibration, achieving error rates of 9% (0.4 m/s) and 10.7% (0.24 m/s) respectively, our results show a similar error margin of 0.7 ± 9.5% (was 0.01 ± 0.33 m/s) for resultant speed. Overall, MBIM with model calibration can capture the impact of the head kinematics during participant falls with reasonable accuracy compared to methods in the literature without the limitations of needing a calibrated space or multiple-camera views required by other methods (Appendix). This attribute renders MBIM with model calibration method particularly beneficial for applications without calibration or multiple-camera views.

The MBIM method relies on accurate image matching for calibration, which involves aligning the known-sized headform with the head being tracked to define the spatial scale. Due to the sensitivity of MBIM to tracking variance, the method includes repetitive trial tracking until the standard error is reduced to below 0.25 m/s. This iterative process contributed to a high interrater reliability (ICC = 0.94), demonstrating strong agreement between raters. However, individual tracking results remain sensitive to slight variations, so relying on a single trial for results is not recommended. Instead, determining a standard error threshold suitable for the MBIM tracking application is critical. A scaling sensitivity analysis revealed that a 10% change in scale while maintaining the same position would result in an approximate 0.2 m/s change in velocity, highlighting the importance of precise scale matching and the value of the MBIM system’s micro-adjustment capability. Inaccurate matching, particularly in rotational alignment, can further introduce scaling errors as the system compensates for rotational discrepancies. However, sensitivity to positional differences depends on camera resolution and distance. Given MBIM’s reliance on accurate matching, it is crucial that trackers are well-trained to ensure consistent and precise feature alignment. Trackers should also practice with representative videos to refine their skills and familiarize themselves with the matching process. This training process can also incorporate feedback loops during training, which can further improve accuracy and consistency in tracking.

This study has several limitations. First, we did not directly compare alternative methods (e.g., Kinovea, Point Click, Multi-Camera) for extracting head impact kinematics, relying instead on literature-reported values for comparison. Additionally, we did not assess the impact of camera resolution or lighting scenarios on tracking accuracy, which may limit the applicability of our results to scenarios involving lower-resolution cameras or poor lighting. However, poor lighting or low resolution would make it harder for trackers to correctly decipher the position of an individual head in a video to perform an accurate match. Another aspect that was not investigated was the effect of frame rate on the accuracy of MBIM. However, previous studies suggest that the content of body movements during falls typically ranges from 1–10 Hz, which can be accurately captured at 30 Hz video frame rates [9, 10]. Bailey et al. suggested that low frame rates, 60 frames per second, are only beneficial in assessing pre-impact velocity. Our study did not verify this MBIM method’s ability to track peak speeds accurately, and we advise caution when using this method with low capture rates for recording peak head speeds during an event. Additionally, impact calculations were computed assuming the timestep between frames remained constant. While the video framerate was verified through the MBIM software, timesteps between frames could fluctuate due to the video encoding process or throttling in the recording device. Another limitation is that the accuracy of MBIM in tracking angular or rotational velocities of the head during impacts was not evaluated, and further analysis is needed to validate the MBIM software for angular and rotational velocity.

Pre-processing of videos can potentially improve and enhance the utility of this MBIM method, while mitigating some video quality limitations. One approach to address the impact of video quality is the application of pre-image processing filters to improve contrast, reduce noise, and enhance the visibility of key features in low-quality videos. Additionally, advanced algorithms, such as motion deblurring techniques or deep learning-based video enhancement, could mitigate motion blur’s effects, particularly in high-speed scenarios. These enhancements could improve tracking accuracy in a broader range of video conditions.

Our results demonstrate that MBIM with model calibration can successfully track the head impact kinematics from single-camera footage without calibration and identify error ranges for different scenarios with varying camera positions and orientations. This development provides an excellent opportunity to capture head impact kinematics without using multiple views or calibration, which other methods require. Therefore, this method could contribute to a better understanding of head impact kinematics and facilitate the development of effective prevention strategies. Regarding real-world applications, MBIM has significant potential for tracking head impact kinematics in dynamic settings, such as sporting events or falls captured on video. MBIM is particularly useful for retrospective analysis of videos where only a single-camera angle is available with no calibration information, like videos posted on sharing platforms or security camera footage. Additionally, MBIM is versatile and capable of tracking other objects, provided a to-scale model of the object is available. It is important to note that the acceptability of errors from this system will depend on the research question of those implementing it, which should be considered before use.

MBIM with model calibration can successfully track the head impact kinematics from single-camera footage without calibration. These results identify error ranges for scenarios with varying camera positions and orientations. This development provides an excellent opportunity to capture head impact kinematics without using multiple views or calibration, which other methods require. Therefore, this method could contribute to a better understanding of head impact kinematics and facilitate the development of effective prevention strategies. However, it is important to note that the acceptability of errors from this system will depend on the research question of those implementing it, which should be considered before use.

## Electronic supplementary material

Below is the link to the electronic supplementary material.

Supplementary file1 (PPTX 14023 kb)
