# Supplementary material for: Uncalibrated Single-Camera View Video Tracking of Head Impact Speeds Using Model-Based Image Matching
Source: Ann Biomed Eng. 2025 Mar 13;53(6):1359–69. doi: 10.1007/s10439-025-03705-2 (PMC12075344; doi:10.1007/s10439-025-03705-2)
Supplement: Supplementary file 1 — Supplementary file1 (PPTX 14023 kb) [file 10439_2025_3705_MOESM1_ESM.pptx]

## Slide 1
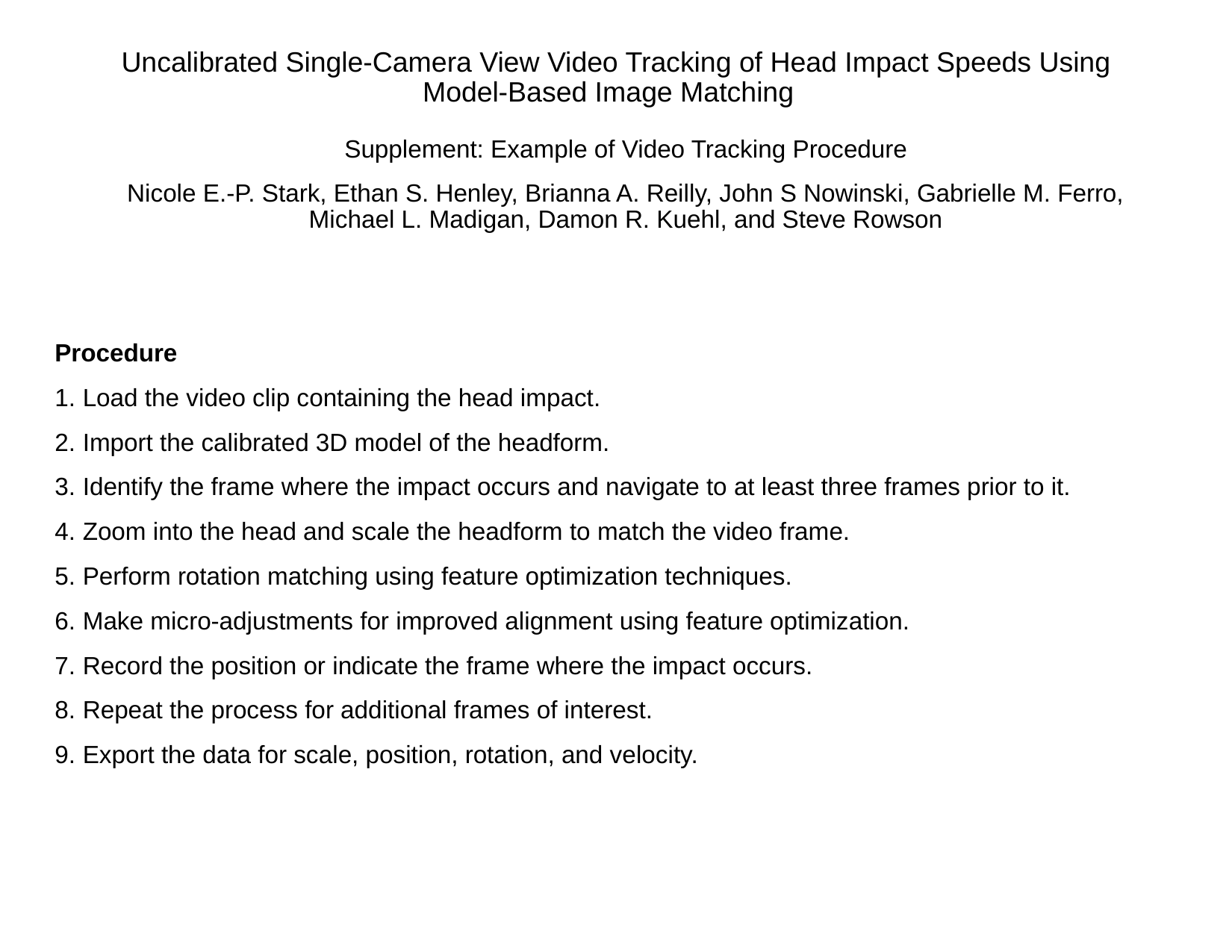

# Uncalibrated Single-Camera View Video Tracking of Head Impact Speeds Using Model-Based Image Matching
Supplement: Example of Video Tracking Procedure
Nicole E.-P. Stark, Ethan S. Henley, Brianna A. Reilly, John S Nowinski, Gabrielle M. Ferro, Michael L. Madigan, Damon R. Kuehl, and Steve Rowson
Procedure
Load the video clip containing the head impact.
Import the calibrated 3D model of the headform.
Identify the frame where the impact occurs and navigate to at least three frames prior to it.
Zoom into the head and scale the headform to match the video frame.
Perform rotation matching using feature optimization techniques.
Make micro-adjustments for improved alignment using feature optimization.
Record the position or indicate the frame where the impact occurs.
Repeat the process for additional frames of interest.
Export the data for scale, position, rotation, and velocity.

## Slide 2
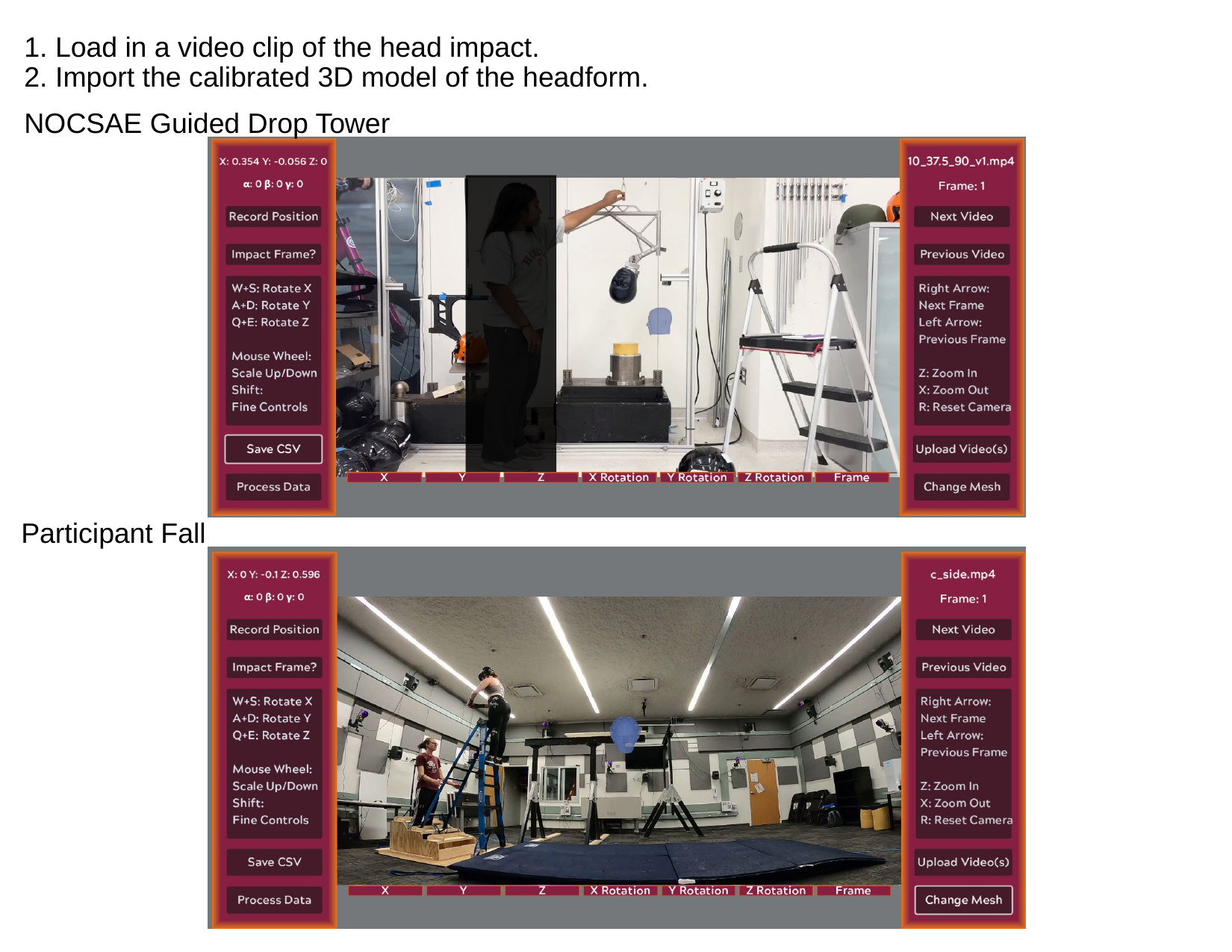

# 1. Load in a video clip of the head impact. 2. Import the calibrated 3D model of the headform.
NOCSAE Guided Drop Tower
Participant Fall

## Slide 3
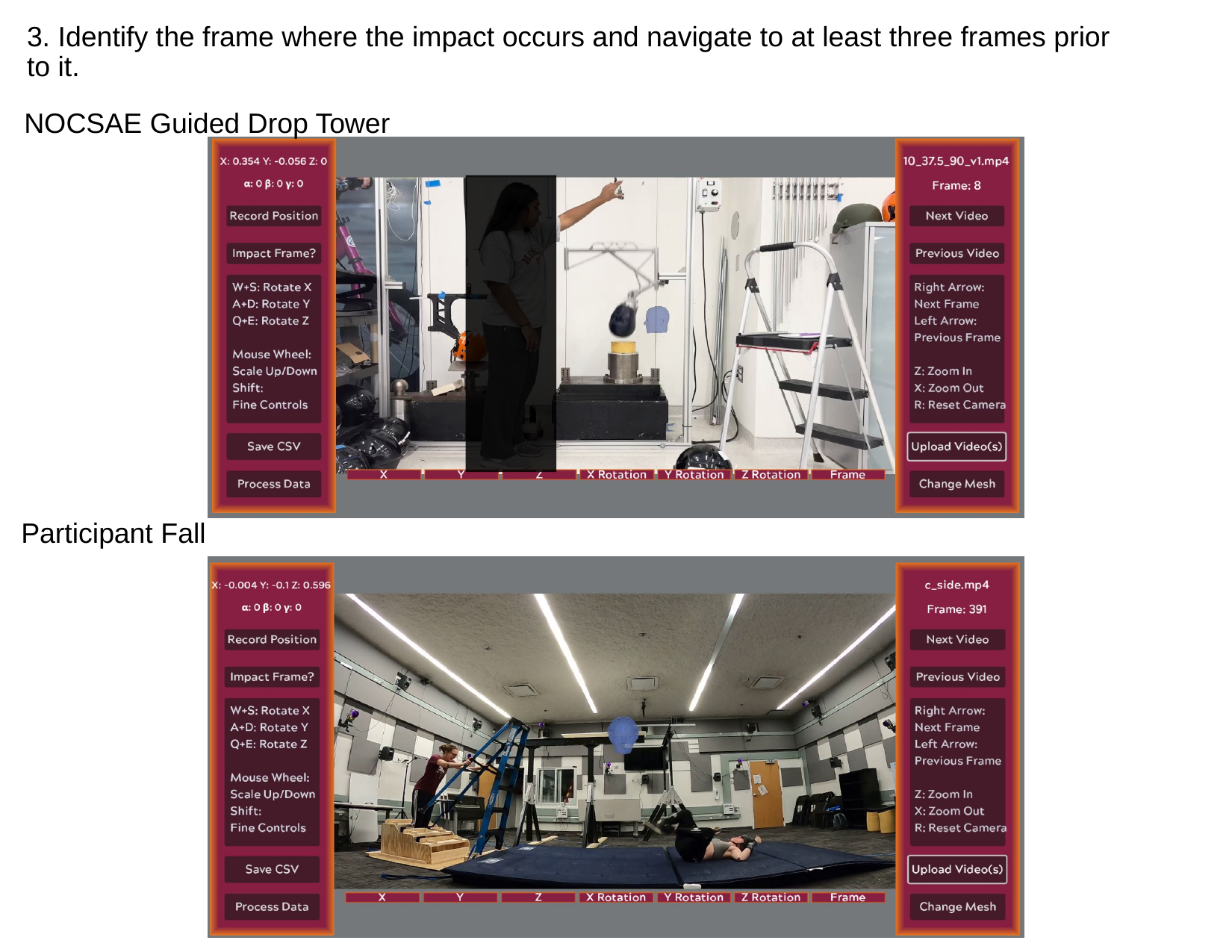

# 3. Identify the frame where the impact occurs and navigate to at least three frames prior to it.
NOCSAE Guided Drop Tower
Participant Fall

## Slide 4
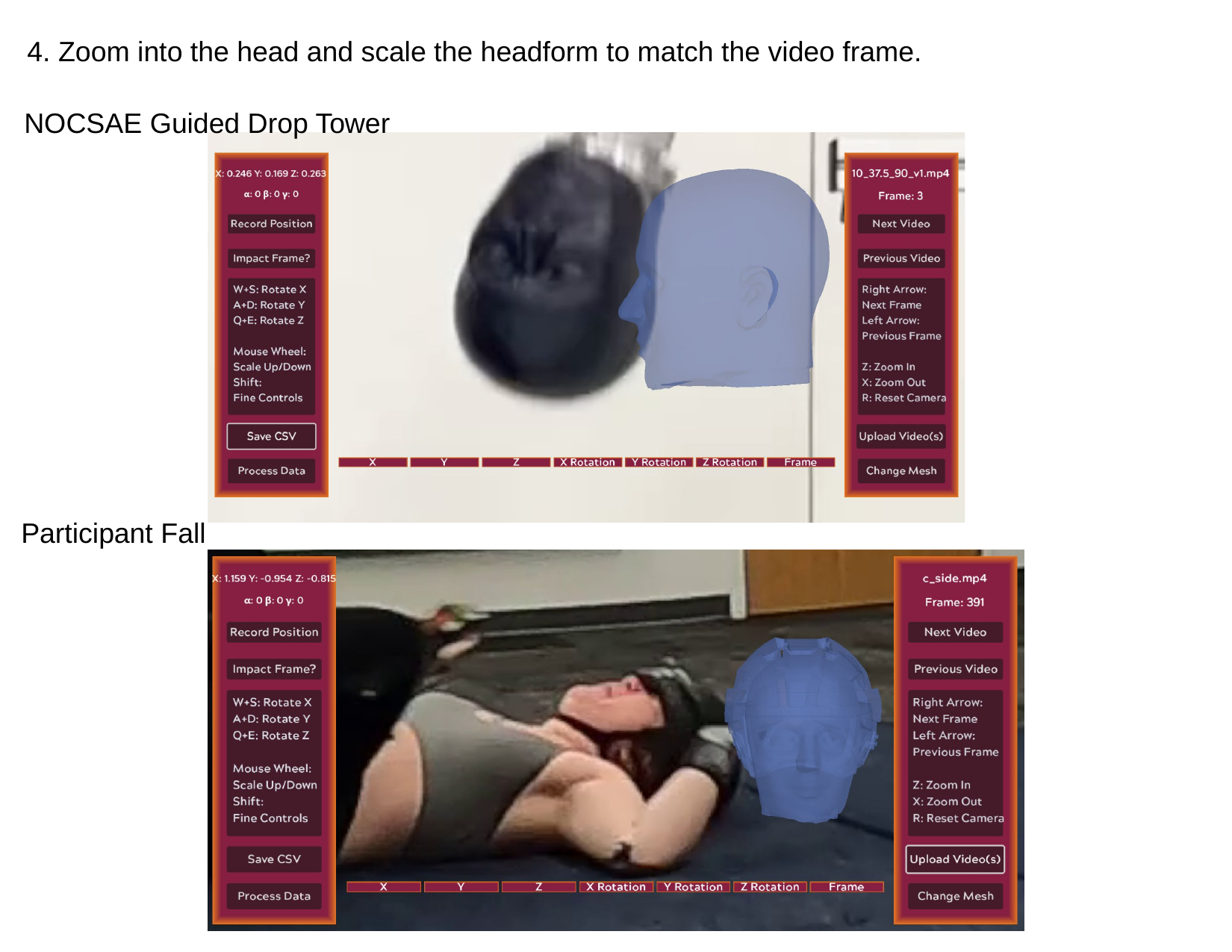

# 4. Zoom into the head and scale the headform to match the video frame.
NOCSAE Guided Drop Tower
Participant Fall

## Slide 5
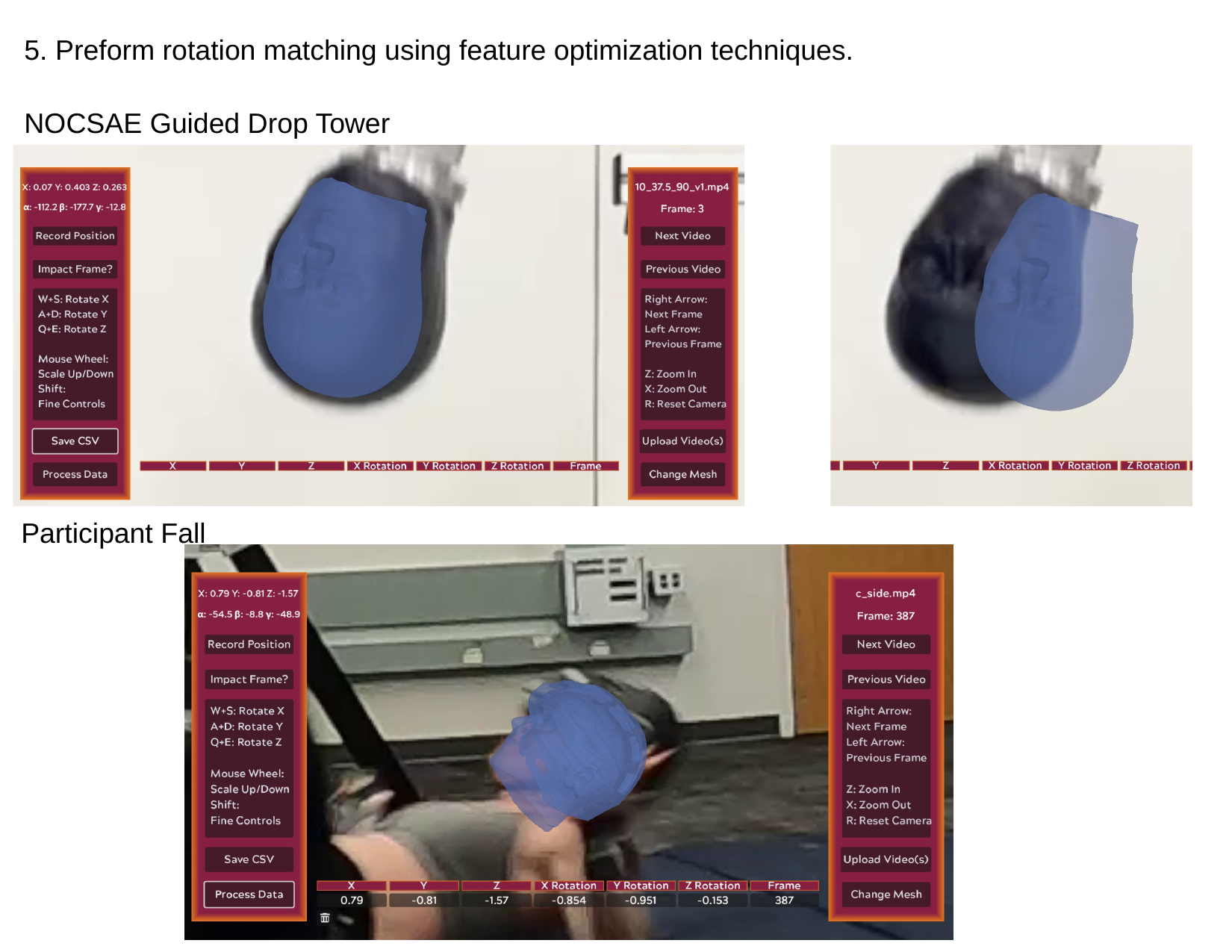

5. Preform rotation matching using feature optimization techniques.
NOCSAE Guided Drop Tower
Participant Fall

## Slide 6
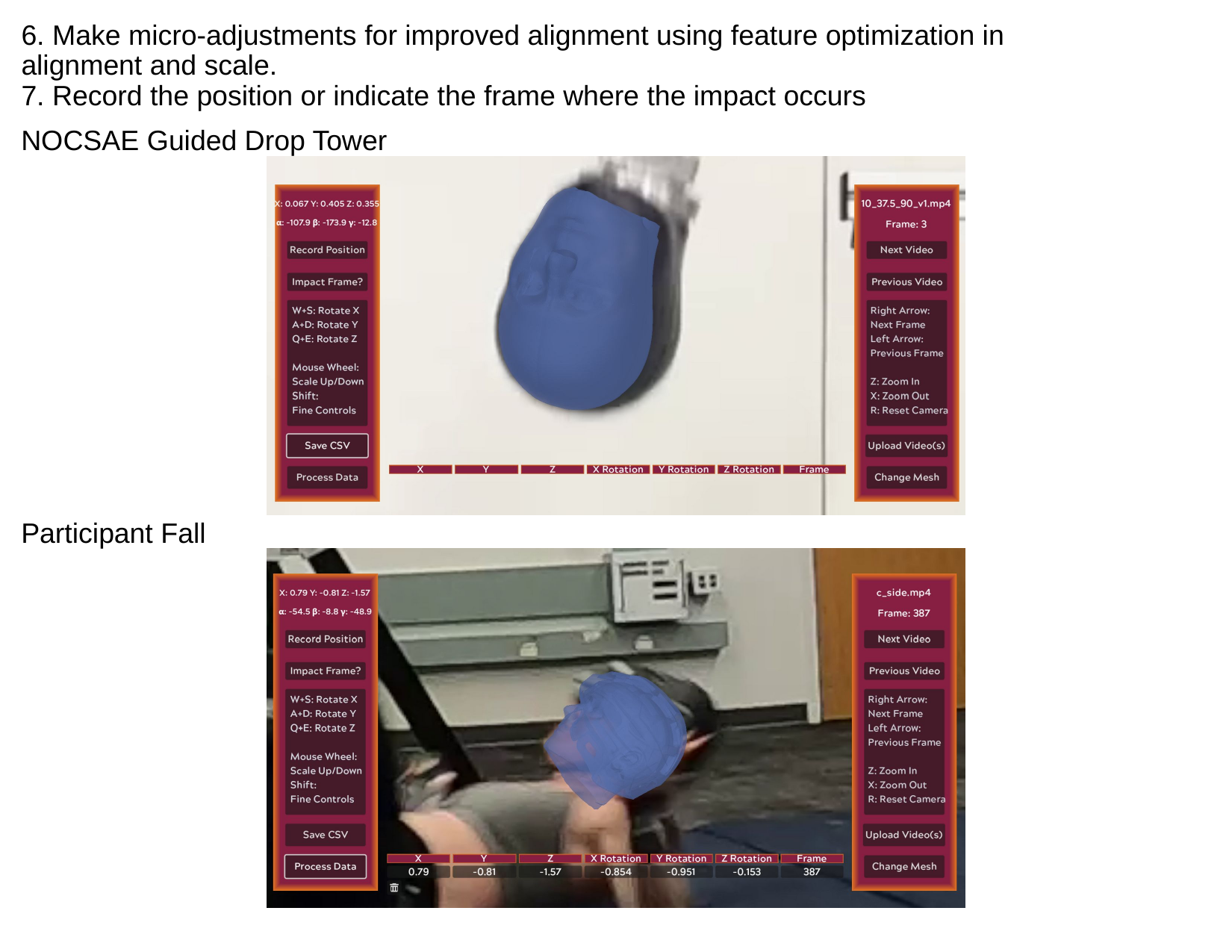

# 6. Make micro-adjustments for improved alignment using feature optimization in alignment and scale. 7. Record the position or indicate the frame where the impact occurs
NOCSAE Guided Drop Tower
Participant Fall

## Slide 7
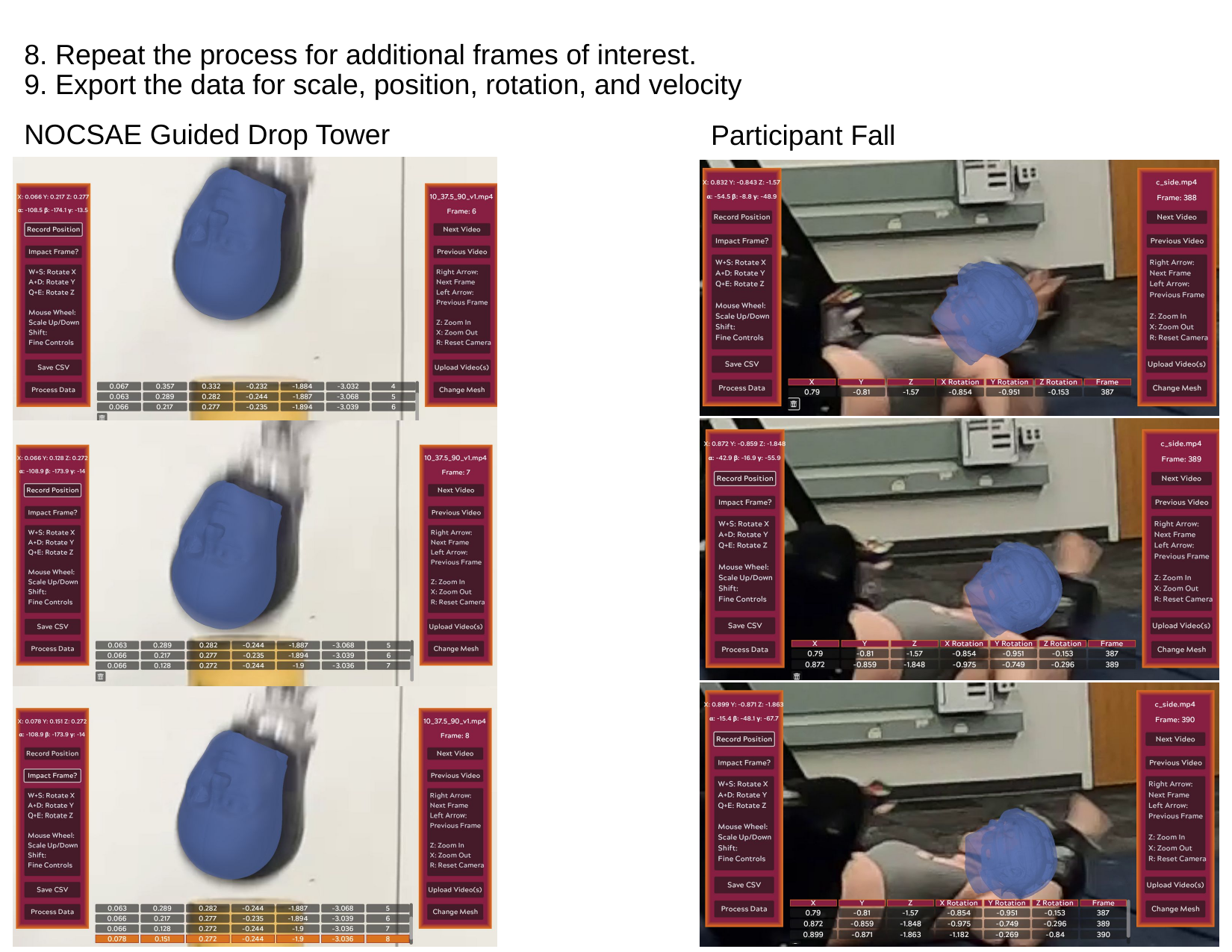

8. Repeat the process for additional frames of interest.
9. Export the data for scale, position, rotation, and velocity
NOCSAE Guided Drop Tower
Participant Fall
